# Prevalence of Proximal Overhangs in Class II Composite Restorations Placed by Undergraduate Students at Qassim University

**DOI:** 10.7759/cureus.96007

**Published:** 2025-11-03

**Authors:** Abdulmajeed N Alwably, Bilal Arjumand, Hamad Mohammad Alharkan, Lama AlJafn, Abdullah F Almoshwih, Asim A Alfawzan

**Affiliations:** 1 College of Dentistry, Qassim University, Buraydah, SAU; 2 Department of Conservative Dental Sciences, College of Dentistry, Qassim University, Buraydah, SAU; 3 Department of Conservative Dental Sciences and Endodontics, College of Dentistry, Qassim University, Buraydah, SAU

**Keywords:** class ii restoration, overhang, qassim university, radiograph, undergraduate dental student

## Abstract

Background

Proximal overhangs in class II composite restorations are common technical errors that can contribute to plaque retention and localized periodontal inflammation. This study aimed to assess the prevalence of proximal overhangs in class II composite restorations placed by undergraduate dental students at Qassim University, Buraydah, Saudi Arabia, using bitewing radiographic analysis.

Methods

A retrospective cross-sectional study was conducted at the College of Dentistry, Qassim University. Competency sheets from fourth- and fifth-year students during the 2023-2024 academic year were reviewed. Bitewing radiographs meeting inclusion criteria (vital permanent teeth with good-quality images) were analyzed. Exclusion criteria included missing adjacent teeth and third molars. Two calibrated evaluators assessed each radiograph with high inter- and intra-examiner reliability (Cohen’s kappa = 0.90). Data were analyzed using SPSS Version 23, and chi-square tests were applied (p < 0.05).

Results

Of the 292 restorations reviewed, 84 (28.8%) met inclusion criteria. Among these, 21 (25%) showed proximal overhangs. Fifth-year students recorded 14 (28%) overhangs versus 7 (20.5%) among fourth-year students (p = 0.793). Female students showed 7 (26%) overhangs compared to 14 (24.6%) for males (p = 0.892). Overhangs were slightly more frequent in maxillary 10 (26.3%) than mandibular 11 (24%) restorations (p = 0.800). Mesial surfaces exhibited 12 (27.9%) overhangs compared to 9 (22%) on distal surfaces (p = 0.529). No associations were statistically significant.

Conclusion

One in four class II composite restorations placed by undergraduate students at Qassim University showed proximal overhangs, with no significant difference by academic year, gender, arch, or surface. The findings highlight a continued need to strengthen matrix adaptation and finishing techniques in undergraduate restorative training.

## Introduction

Dental restorations play a crucial role in preserving a tooth's form, function, and aesthetics while preventing recurrent caries and periodontal diseases. Research indicates that excessively bulky or irregular restorations may contribute to periodontal issues by promoting bacterial plaque accumulation rather than causing mechanical irritation. Epidemiological and clinical studies have highlighted the link between such iatrogenic factors and the development of local periodontal problems [1-7]. Inadequate dental restorations and prostheses frequently lead to gingival inflammation and periodontal damage [8].

Despite advancements in restorative dentistry and dental materials, it appears that restorations with overhang margins persist, occasionally going unnoticed or unaddressed. This could be a noteworthy contributor to the development of periodontal disease [3]. Thorough examination, combining clinical and radiographic assessments, is the most reliable method for identifying problematic overhanging margins [4]. The main local factor associated with periodontal disease in adults is overhanging dental restorations. This occurs when the restorative material extends beyond the boundaries of the cavities [9].

Examining contact points and posterior tooth areas for carious lesions or overhanging restorations is challenging using conventional clinical methods. Bitewing radiographs have been shown to be more effective in detecting proximal lesions and identifying insufficiently treated filled surfaces compared to relying solely on clinical examination [10].

Faulty restoration techniques and variations in the cervical aspect of the tooth, encompassing features such as furcation, fluting, and concavities, play a role in suboptimal restoration with overhang. This complicates the consistent placement of a wedge and matrix band for complete adaptation to the gingival cavomargin. Overhangs in restoration, termed as permanent calculus, lead to issues such as plaque accumulation, caries, and periodontal disease [11].

The aim of this study is to investigate the prevalence of proximal overhangs in class II composite restorations performed by undergraduate students at Qassim University, Buraydah, Saudi Arabia. Since there is a lack of data on the occurrence of overhangs in restorations placed by undergraduate students, this study provides valuable information to address this gap.

## Materials and methods

Ethical approval

The study was approved by the Committee of Research Ethics at Qassim University under reference number (24-71-11). Approval was also obtained from Medical City at Qassim University.

Study design

This is a retrospective cross-sectional study of class II restoration performed by undergraduate students at the College of Dentistry, Qassim University. Data were collected from competency sheets for fourth- and fifth-year students (2023-2024 academic year), and subsequently for selected cases, radiographs were collected according to inclusion criteria and exclusion criteria.

All radiographic assessments were performed using digital bitewing radiographs retrieved from the university’s clinical database. Radiographs were reviewed on a calibrated monitor under standardized viewing conditions. Images showing cone-cutting, overlapping proximal surfaces, or distortion were excluded to minimize radiographic errors. Overhangs were assessed on the radiographs by two calibrated examiners (restorative specialists) using image analysis software with zoom and contrast adjustment features. A restoration was classified as having an overhang if the restorative material extended beyond the radiographic outline of the cavity margin, forming a radiopaque projection into the proximal space. Calibration sessions were conducted before data collection, and inter- and intra-examiner reliability were confirmed (Cohen’s κ = 0.90).

Only radiographs of vital permanent teeth with good image quality were included. Cases involving third molars were excluded, as well as those where an adjacent tooth was missing.

Data collection

Out of 292 class II restorations done in one academic year, 84 cases were included according to inclusion and exclusion criteria. Data were entered into Excel (Microsoft Corp., Redmond, WA), including patient file, student academic year, whether done by male or female student, mesial or distal, upper or lower, and if there is overhang restoration or not.

Data management and analysis method

The collected data were handled and kept for record-keeping in Primedent software in Dental clinics at Qassim University. The data were analyzed using Statistical Package for Social Sciences Version 23 (IBM Corp., Armonk, NY), and the comparison between groups was conducted using the chi-square test, with the significance level set at p < 0.05.

## Results

Out of 84 class II restorations with radiographs, 21 (25%) cases showed overhangs. Fifth-year students had a higher percentage of overhangs, 14 (28%), compared to fourth-year students, 7 (20%) (p = 0.793). Female students showed slightly more overhangs, 7 (26%), than male students, 14 (24.6%) (p = 0.892). Overhangs were more common in upper restorations, 10 (26.3%), than in lower restorations, 11 (24%) (p = 0.800). Mesial surfaces had more overhangs, 12 (27.9%), compared to distal surfaces, 9 (22%) (p = 0.529). The chi-square test results revealed that none of the observed differences were statistically significant. Interexaminer reliability was excellent, with Cohen’s kappa = 0.9, confirming consistency in radiographic assessment (Table 1).

**Table 1:** Prevalence of overhangs by student year, gender, jaw, and tooth surface

Group	Total (n)	Overhang (n)	Percentage	Chi-square	P-value
Student year	Fifth year (n=54)	14	28%	0.069	0.793
	Fourth year (n=30)	7	20%		
Gender	Male (n=57)	14	24.6%	0.018	0.892
	Female (n=27)	7	26%		
Jaw	Upper (n=38)	10	26.3%	0.064	0.800
	Lower (n=46)	11	24%		
Tooth surface	Mesial (n=43)	12	27.6%	0.397	0.529
	Distal (n=41)	9	22.7%		

## Discussion

In this study, one-quarter (25%) of class II composite restorations placed by undergraduate students at Qassim University demonstrated proximal overhangs. This finding highlights that, despite advances in restorative techniques and materials, overhangs remain a common technical challenge in dental education.

Similar studies conducted at other institutions have reported comparable or even higher prevalence rates. For instance, a study conducted by AlOtaibi et al. in Riyadh found that 13% of student restorations had overhangs, identifying it as the most frequent defect among class II composites [12]. Another study by Quadir et al., conducted at Fatima Jinnah Dental College in Karachi, reported a prevalence of overhanging restorations is 58% among undergraduate students [13]. Another study conducted by Kamel and Salman at Tishk University also explored the prevalence of overhangs in class II restorations among undergraduate students and reported a 20% prevalence of overhangs [14]. Another study by Balhaddad et al. conducted at Imam Abdulrahman Bin Faisal University reported a 9.96% prevalence of overhangs in class II composite restorations placed by undergraduate students [15]. These variations may reflect differences in teaching protocols, operator supervision, or evaluation methods, but together they reinforce that overhangs are consistently encountered in undergraduate clinics worldwide.

Our results also showed that fifth-year students placed slightly more overhanging restorations (28%) than fourth-year students (20.5%), though this difference was not statistically significant. Likewise, no significant differences were observed between male and female students, between upper and lower arches, or between mesial and distal surfaces. These findings suggest that overhang formation is not strongly influenced by seniority or gender but rather by the inherent difficulty of achieving proper matrix adaptation and contour in proximal restorations.

The persistence of overhangs across studies and institutions underscores the complexity of restoring proximal surfaces. Inadequate matrix band placement, poor wedge adaptation, and limited visibility in posterior teeth all contribute to this outcome. Even with careful finishing and polishing, small undetected overhangs may remain and are best identified radiographically.

Clinically, overhanging margins are more than just technical imperfections; they act as plaque-retentive areas that can lead to periodontal inflammation and recurrent caries if not corrected. Educationally, these findings suggest the need for reinforcing matrix and wedge placement techniques, encouraging the use of newer matrix systems, and strengthening supervision during clinical sessions. Simulation-based training followed by immediate clinical feedback may also help reduce the prevalence of these iatrogenic errors.

Considering the limitations of this study, which include relying solely on radiographic evaluation without clinical examination and restricting the sample to one academic year, the observed 25% prevalence of overhangs offers valuable local insight. These findings are consistent with results from other dental schools and emphasize that overhangs remain a persistent challenge in restorative dental education. This highlights the ongoing need to strengthen undergraduate training in matrix adaptation, wedging, and finishing techniques.

## Conclusions

This study found that one in four class II composite restorations placed by undergraduate students presented proximal overhang. The absence of statistically significant differences between student year, gender, arch, or surface suggests that overhangs are a widespread technical challenge rather than being limited to specific groups. These findings underscore the importance of reinforcing teaching strategies related to matrix adaptation, wedging, and finishing techniques in undergraduate programs. By addressing these issues during training, dental schools can help ensure that future practitioners deliver restorations that are both functional and biologically sound.

## References

[1] Tervonen T, Ainamo J (1986). Relative influence of calculus and overhangs of fillings on the frequency of score 2 of the CPITN. Community Dent Oral Epidemiol.

[2] Hakkarainen K, Ainamo J (1980). Influence of overhanging posterior tooth restorations on alveolar bone height in adults. J Clin Periodontol.

[3] Gilmore N, Sheiham A (1971). Overhanging dental restorations and periodontal disease. J Periodontol.

[4] Jansson L, Ehnevid H, Lindskog S, Blomlöf L (1994). Proximal restorations and periodontal status. J Clin Periodontol.

[5] Pack AR, Coxhead LJ, McDonald BW (1990). The prevalence of overhanging margins in posterior amalgam restorations and periodontal consequences. J Clin Periodontol.

[6] Than A, Duguid R, McKendrick AJ (1982). Relationship between restorations and the level of the periodontal attachment. J Clin Periodontol.

[7] Rodriguez-Ferrer HJ, Strahan JD, Newman HN (1980). Effect of gingival health of removing overhanging margins of interproximal subgingival amalgam restorations. J Clin Periodontol.

[8] Al-Hamdan KS (2008). Prevalence of overhang interproximal amalgam restorations. PODJ.

[9] Sikri VK, Sikri P (1993). Overhanging interproximal silver amalgam restorations. Prevalence and side-effects. Indian J Dent Res.

[10] Kells BE, Linden GJ (1992). Overhanging amalgam restorations in young adults attending a periodontal department. J Dent.

[11] Nicholsen JW (2006). Biologic considerations. Fundamentals of Operative Dentistry: A Contemporary Approach.

[12] AlOtaibi GL, Aldakheel R, Alhussein H, Alrowili S (2020). Outcomes of class II composite restorations placed by dental students: an observational study. Saudi J Oral Sci.

[13] Quadir F, Ali Abidi SY, Ahmed S (2014). Overhanging amalgam restorations by undergraduate students. J Coll Physicians Surg Pak.

[14] Kamel JH, Salman FD (2023). The prevalence of overhang and gap formation in posterior amalgam and composite restorations among 4th and 5th stage undergraduate students/Tishk University. Acta Sci Med Sci.

[15] Balhaddad AA, AlGhamdi N, Alqahtani M, Alsulaiman OA, Alshammari A, Farraj MJ, Alsulaiman AA (2024). Predictors of procedural errors in class II resin composite restorations using bitewing radiographs. Saudi Dent J.

